# Supplementary material for: Nanopore analysis of salvianolic acids in herbal medicines
Source: Nat Commun. 2024 Mar 5;15:1970. doi: 10.1038/s41467-024-45543-1 (PMC10915175; doi:10.1038/s41467-024-45543-1)
Supplement: Supplementary file 1 — Supplementary Information [file 41467_2024_45543_MOESM1_ESM.pdf]

Supplementary Information for

Nanopore analysis of salvianolic acids in herbal medicines

Pingping Fan,[ab#] Shanyu Zhang,[ab#] Yuqin Wang,[abcd] Tian Li, [ab] Hanhan Zhang, [ab] Panke Zhang,[a] and Shuo Huang[*ab]

[a.] State Key Laboratory of Analytical Chemistry for Life Sciences, School of Chemistry and Chemical Engineering, Nanjing University, 210023, Nanjing, China.
[b.] Chemistry and Biomedicine Innovation Center (ChemBIC), Nanjing University, 210023, Nanjing, China.
[c.] State Key Laboratory of Pollution Control and Resource Reuse, School of the Environment Nanjing University, 210023, Nanjing, China.
[d.] Institute for the Environment and Health, Nanjing University Suzhou Campus, 215163, Suzhou, China

* Corresponding author. Email: shuo.huang@nju.edu.cn (S.H.);

Materials

Potassium chloride (KCl), 3-(N-Morpholino) propane sulfonic acid (MOPS), salvianic acid A, rosmarinic acid and salvianolic acid A were from Aladdin (China). Caffeic acid and protocatechualdehyde were from ACMEC. Protocatechuic acid was from Shanghai Yuanye Bio-Technology. Lithospermic acid, salvianolic acid B and *trans*-1,2-cyclohexanediol were from Macklin (China). *E. coli* BL21 (DE3) pLysS was from Sangon Biotech. 3-(maleimide) phenylboronic acid (MPBA, Cat. #sc-352346) was from Santa Cruz Biotechnology (Shanghai) Co., Ltd. Hexadecane, pentane and Genapol X-80 were purchased from Sigma-Aldrich. Glycerol, dioxane-free isopropyl-β-D-thiogalactopyranoside (IPTG), kanamycin sulfate, imidazole and tris (hydroxymethyl)aminomethane (Tris) were from Solarbio. SDS-PAGE electrophoresis buffer powder was from Beyotime. Precision Plus ProteinTM Dual color Standards, TGXTM FastCastTM Acylamide Kit (4-15%), stacking gel buffer (0.5 M Tris-HCl buffer, pH 6.8) and resolving gel buffer (1.5M Tris-HCl buffer, pH 8.8) were obtained from Bio-Rad. 1,2-diphytanoyl-sn-glycero-3-phosphocholine (DPhPC) was from Avanti Polar Lipids.

The potassium chloride buffer (1.5 M KCl, 100 mM MOPS, pH 7.0) was prepared with Milli-Q water and membrane (0.2 µm, Whatman) filtered prior to use. The stock solutions of caffeic acid, salvianic acid A, protocatechuic acid, protocatechualdehyde, rosmarinic acid, lithospermic acid, salvianolic acid B and *trans*-1,2-cyclohexanediol in Milli-Q water were prepared with a 100 mM concentration. The stock solution of salvianolic acid A was prepared in the Milli-Q water with a 50 mM concentration. The salvianolate injection was purchased from Green Valley (Shanghai) Pharmaceutical Technology Co., Ltd. *Salvia miltiorrhiza* was purchsed from Juxian, Shandong Province of China. *Rosemary* and *P. vulgaris* were purchased from Bozhou City, Anhui province of China.

Supplementary Table 1. The statistics of $\mathbf{1/\tau_{on}}$ and $\boldsymbol{\tau_{off}}$ of SalB. All measurements were performed as described in **Methods**. SalB was added to *cis* with a variety of different concentrations. A +100 mV bias was continually applied during the measurements. The $\overline{1/\tau_{on}}$ and $\overline{\tau_{off}}$ were mean values of $1/\tau_{on}$ and τ_{off} acquired from three independent measurements**.**

Salvianolic Acids	Concentration (μM)	$\overline{1/\tau_{on}}$ (ms^{-1})	$\overline{\tau_{off}}$ (ms)
	25	$(4.3 \pm 0.8) * 10^{-4}$	$(0.86 \pm 0.04) * 10^{3}$
	50	$(8.3 \pm 0.5) * 10^{-4}$	$(0.8 \pm 0.07) * 10^{3}$
SalB	75	$(1.14 \pm 0.07) * 10^{-3}$	$(0.83 \pm 0.16) * 10^{3}$
	100	$(1.63 \pm 0.06) * 10^{-3}$	$(0.83 \pm 0.10) * 10^{3}$
	125	$(2.28 \pm 0.09) * 10^{-3}$	$(0.85 \pm 0.10) * 10^{3}$

Supplementary Table 2. Ratios of events removed by computer algorithms. Interference events generated during the acquisition with salvianolic acids and salvianolate injection were removed by DBSCAN algorithms. Interference events generated during the acquisition with natural herbs were removed by One-Class SVM. The ratio of removed events is summarized below.

Salvianolic Acids and Natural Samples	Ratio of interference events (%)
CA	5.7 ± 1.3
PCA	5 ± 2
PA	2.9 ± 0.9
SAA	18.3 ± 1.9
RA	10.8 ± 1.1
LSA	12.5 ± 0.4
SalA	30 ± 2
SalB	7.1 ± 1.1
salvianolate injection	21.4 ± 0.6
Salvia miltiorrhiza	31 ± 4
Rosemary	70 ± 4
P. vulgaris	78 ± 6

Supplementary Table 3. The $\Delta I/I_o$ and $S.D.$ of binding events of different salvianolic acids. Nanopore events of different salvianolic acids were separately acquired, analyzed and demonstrated. All measurements were performed as described in **Methods** and a +100 mV bias was continually applied during the measurements. All statistical results were derived from results acquired from three independent measurements (N=3).

Salvianolic Acids	type Ⅰ $\Delta I/I_o$	type Ⅱ $\Delta I/I_o$	type Ⅲ $\Delta I/I_o$
CA	(1554 ± 6) * 10^{-4}	—	—
PCA	(1252 ± 3) * 10^{-4}	—	—
PA	(1878 ± 4) * 10^{-4}	—	—
SAA	(1296 ± 4) * 10^{-4}	(2538 ± 4) * 10^{-4}	—
RA	(1141 ± 12) * 10^{-4}	(297 ± 5) * 10^{-3}	—
LSA	(984 ± 9) * 10^{-4}	(2049 ± 7) * 10^{-4}	—
SalA	(2449 ± 5) * 10^{-4}	(355 ± 3) * 10^{-3}	(417 ± 3) * 10^{-3}
SalB	(97 ± 2) * 10^{-3}	(331 ± 3) * 10^{-3}	(345 ± 5) * 10^{-3}

Salvianolic Acids	type Ⅰ $S.D.$ (pA)	type Ⅱ $S.D.$ (pA)	type Ⅲ $S.D.$ (pA)
CA	1.504 ± 0.015	—	—
PCA	1.622 ± 0.006	—	—
PA	1.246 ± 0.006	—	—
SAA	1.27 ± 0.11	8.46 ± 0.10	—
RA	2.465 ± 0.019	7.15 ± 0.08	—
LSA	3.894 ± 0.018	4.60 ± 0.06	—
SalA	5.6 ± 0.2	2.42 ± 0.18	3.44 ± 0.12
SalB	2.59 ± 0.05	9.6 ± 0.2	22.26 ± 0.17

Supplementary Table 4. Quantitative analysis of salvianolate injection. All measurements were performed as described in **Methods**. A +100 mV bias was continually applied during the measurements. To initiate the measurement, the salvianolate injection was added to the *cis* chamber with a final concentration of 0.04 mg/mL (**Fig. 4**). The $1/\tau_{on}$ of SalB acquired with the salvianolate injection was derived as described in **Methods**. The measured values were derived from the calibration curve of SalB (**Supplementary Fig. 3**, **Supplementary Table 1**).

Salvianolate Injection (SalB)	Concentration (mg/mL)	$1/\tau_{on}$ (ms^{-1})	Measured (mg)
trial 1	0.04	$6.00 * 10^{-4}$	35.30
trial 2	0.04	$5.91 * 10^{-4}$	34.82
trial 3	0.04	$5.96 * 10^{-4}$	35.08

Supplementary Table 5. Quantitative analysis of natural herbs. All measurements were performed as described in **Methods**. A +100 mV bias was continually applied. To initiate the measurement, 20 µL filtrate obtained from each herb sample was respectively added to the *cis* chamber (**Fig. 5**). The $1/\tau_{on}$ of SalB acquired with *Salvia miltiorrhiza* and the $1/\tau_{on}$ of RA acquired with *Rosemary* and *P. vulgaris* were respectively derived as described in **Methods**. The measured $1/\tau_{on}$ values were then converted to amount of the target analyte based on the calibration curve of SalB and RA (**Supplementary Figs. 3 and 30**, **Supplementary Tables 1 and 7**).

Natural herbs	$1/\tau_{on}$ (ms^{-1})	Concentration in water extract (µM)	Concentration in herbs (mg/g)
Salvia miltiorrhiza (SalB)	$(5 \pm 2) * 10^{-4}$	$(8 \pm 3) * 10^{2}$	12 ± 4
Rosemary (RA)	$(90 \pm 6) * 10^{-6}$	174 ± 10	1.26 ± 0.08
P. vulgaris (RA)	$(40 \pm 9) * 10^{-6}$	74 ± 16	0.53 ± 0.12

Supplementary Table 6. Comparison between our results and that of relevant literatures. The measured SalB concentration in *Salvia miltiorrhiza* and RA in *Rosemary* and *P. vulgaris* reported in our work and that from previous literatures were compared. In our work, the salvianolic acids content in natural herbs was calculated as described in **Supplementary Table 5** and **Methods**. The corresponding material source, extraction or pretreatment methods were also summarized below.

Natural herbs	Content (mg/g)	Materials source	Methods	References
Salvia miltiorrhiza (SalB)	12 ± 4 (our work)	Juxian, Shandong Province of China	water for extraction, soaking for 16 h	
	4.77~40 (HPLC)	Linqu County, Shandong Province of China	70% aqueous methanol for extraction, ultrasound for 20 min	*Molecules* 17, 2388-2407 (2012)
	17.92~34.8 (HPLC)	Laiwu City, Shandong Province of China	water for extraction, reflux for 4 h	*Herald of Medicine* 38, 1624-1629 (2019)
	~20 (HPLC)	Shangluo, Shaanxi Province of China	water for extraction, ultrasound for 30 min, ethyl acetate reextraction for 5 times	*Ultrasonics Sonochemistry* 17, 61-65 (2010)
Rosemary (RA)	1.26 ± 0.08 (our work)	Bozhou City, Anhui Province of China	water for extraction, soaking for 16 h	
	0.014~6.5 (HPLC)	Peñafiel (Valladolid, Spain)	pretreatment with deoiled, deoiled + milled or fresh leaves, water for extreation at 40 °C for 4 h	*Journal of Food Engineering* 109, 98-103 (2012)
	0.9~41.8 (HPLC-MS)	Murcia in Spain	Methanol extraction	*Journal of Chromatography A* 1120, 221-229 (2006)
P. vulgaris (RA)	0.53 ± 0.12 (our work)	Bozhou City, Anhui Province of China	water for extraction, soaking for 16 h	
	1.19~9.46 (HPLC)	Turkey	water for extraction with HCl and ascorbic acid addition for 8h stirring	*Journal of Pharmaceutical and Biomedical Analysis* 55, 1227-1230 (2011)
	2~7 (HPLC)	China	methanol for extraction, relux for 1 h	*Chinese Traditional and Herbal Drugs* 51, 2842-2850 (2020)

Supplementary Table 7. The statistics of $\boldsymbol{1/\tau_{on}}$ and $\boldsymbol{\tau_{off}}$ of RA. All measurements were performed as described in **Methods**. RA was added to *cis* with varying concentrations. A +100 mV bias was continually applied during the measurements. The $\overline{1/\tau_{on}}$ and $\overline{\tau_{off}}$ were mean values of $1/\tau_{on}$ and τ_{off} acquired from three independent measurements**.**

Salvianolic Acids	Concentration (μM)	$\overline{1/\tau_{on}}$ (ms^{-1})	$\overline{\tau_{off}}$ (ms)
	2	(2.7 ± 0.5) * 10^{-5}	(0.8 ± 0.2) * 10^{3}
	5	(8.6 ± 1.7) * 10^{-5}	(0.95 ± 0.15) * 10^{3}
RA	10	(14.2 ± 1.9) * 10^{-5}	(0.71 ± 0.14) * 10^{3}
	20	(26 ± 6) * 10^{-5}	(0.10 ± 0.09) * 10^{3}
	30	(40 ± 4) * 10^{-5}	(0.89 ± 0.07) * 10^{3}

Supplementary Table 8. The range of effective concentrations. All measurements were performed as described in **Methods**. Each analyte was respectively added to *cis* with varying concentrations (**Supplementary Fig. 31**). A +100 mV bias was continually applied during the measurements. The range of effective concentrations were determined as the range of the input analyte concentration, within which the $\overline{1/\tau_{on}}$ values are linearly correlated with the input analyte concentration. All results were acquired from three independent measurements.

Salvianolic Acids and Natural Samples	The range of effective concentrations (μM)
CA	25 ~ 500
PCA	50 ~ 2000
PA	5 ~ 400
SAA	50 ~ 1500
RA	2 ~ 30
LSA	25 ~ 300
SalA	2 ~ 40
SalB	25 ~ 125

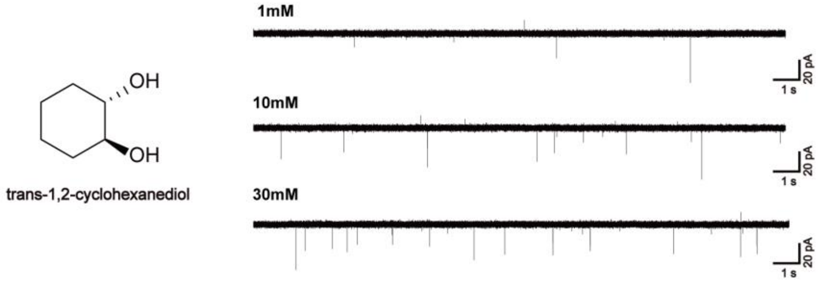

Supplementary Fig. 1. Single molecular sensing of *trans*-1,2-cyclohexanediol. (Left) The chemical structure of *trans*-1,2-cyclohexanediol. **(Right)** The *trans*-1,2-cyclohexanediol sensing performed at different concentrations of analyte using MspA-90PBA. Only extremely short residing events with no well-defined event features were shown, demonstrating that *trans*-diols would fail to report any events when probed by MspA-90PBA.

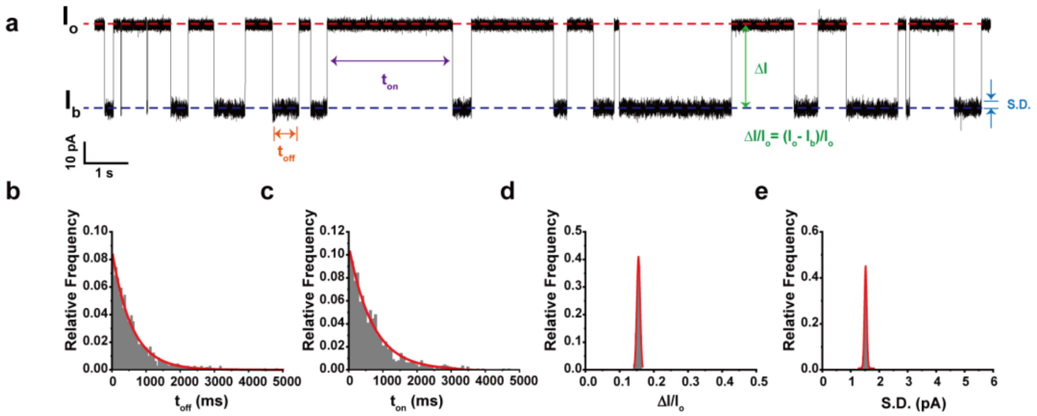

Supplementary Fig. 2. The definition of event parameters. (a) A representative trace containing nanopore events. Here, caffeic acid was applied as the model analyte. I_o is the open pore current and I_b is the residual current of an event. The relative blockage depth $\Delta I/I_o$ is derived from $\Delta I/I_o = (I_o - I_b)/I_o$. $S.D.$ is the standard deviation value of the blockage segment of an event and t_{off} is the dwell time of an event. t_{on} is the inter-event interval. **(b-c)** The event histogram of t_{off} **(b)** and t_{on} **(c)** acquired from a segment of a continually recorded trace. The histograms were single exponentially fitted according to the equation $y = a * exp(-t/\tau)$. **(d-e)** The event histogram of $\Delta I/I_o$ **(d)** and $S.D.$ **(e)** acquired from a segment of a continually recorded trace. The histograms of **d** and **e** were Gaussian fitted according to the equation $y = y_0 + \frac{A}{w\sqrt{\pi/2}} . e^{-2\frac{(x-x_c)^2}{w^2}}$. Unless otherwise stated, all event parameters used in this paper were defined as described above.

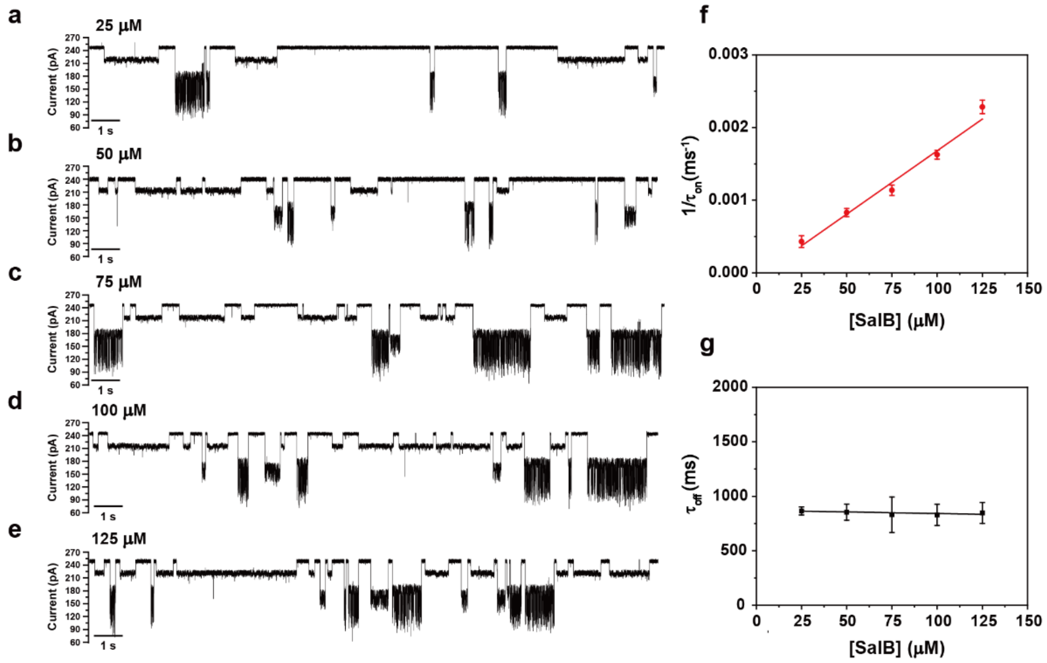

Supplementary Fig. 3. The concentration dependence of SalB sensing. (a-e) Representative traces of SalB sensing performed with different SalB concentrations. All measurements were performed using MspA-90PBA in a buffer of 1.5 M KCl, 100 mM MOPS, pH 7.0 and a +100 mV bias was continually applied (**Methods**). SalB was added to *cis* with a final concentration of 25-100 μM. **(f)** The plot of $1/\tau_{on}$ versus the SalB concentration. The $1/\tau_{on}$ is linearly correlated with the SalB concentration. **(g)** The plot of τ_{off} versus the SalB concentration. The τ_{off} remains constant despite of the change of the SalB concentration. The data in **(f-g)** show mean ± standard deviations derived from results of three independent measurements (N=3). The error bars represent standard deviation values. All statistic results are also demonstrated in **Supplementary Table 1**.

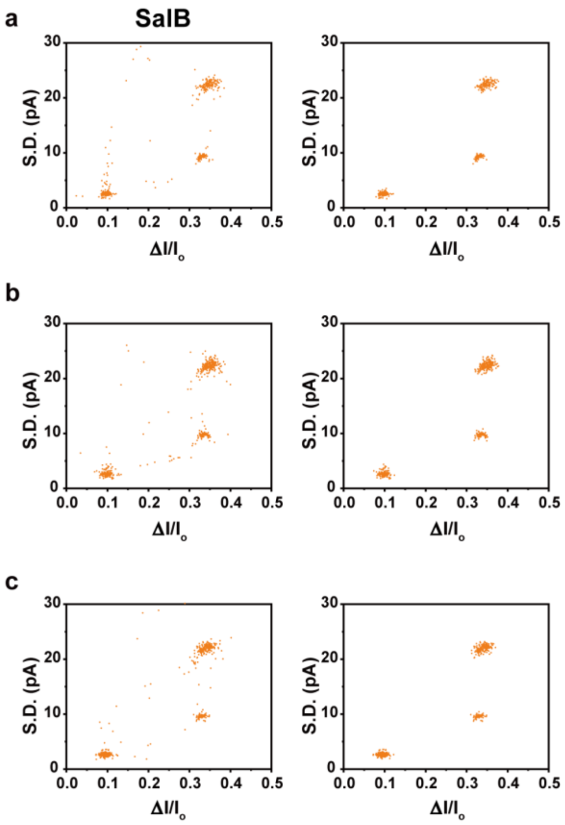

Supplementary Fig. 4. Cluster analysis of SalB. **(Left)** The original scatter plots of $\Delta I/I_o$ versus $S.D.$ of events acquired with SalB. Three independent trials **(a-c)** with identical measurement conditions were separately performed. Specifically, the measurements were performed with MspA-90PBA in a buffer of 1.5 M KCl, 100 mM MOPS, pH 7.0 and a +100 mV bias was continually applied. SalB was added to *cis* with a final concentration of 0.1 mM. Results in each scatter plot were from a 20 min continuously recorded trace. **a)** 730 events, **b)** 840 events and **c)** 826 events were respectively included in each scatter plot. **(Right)** The scatter plots of $\Delta I/I_o$ versus $S.D.$ after cluster analysis treatment. To remove background noise events in the original scatter plots (left), the raw data were further treated by cluster analysis using DBSCAN. The epsilon was set to 0.08 and the min_samples was set to 11. Each right scatter plot demonstrates the same set of data however with the background noise events removed. After the treatment, **a)** 672 events, **b)** 774 events and **c)** 776 events were respectively retained. All statistical results are also demonstrated in **Supplementary Table 3.**

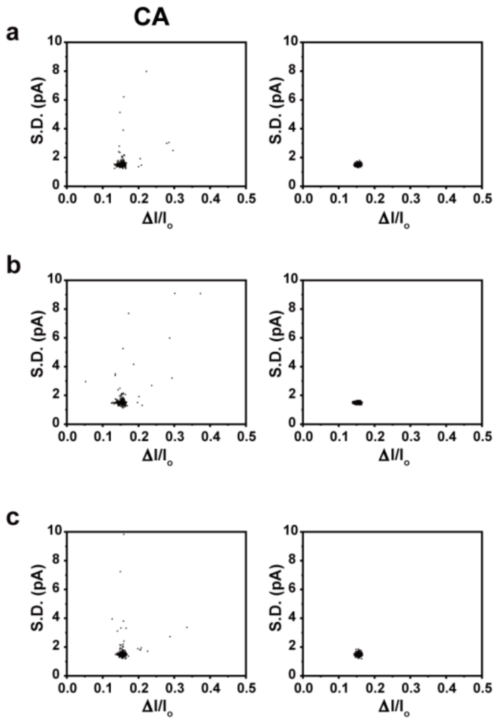

Supplementary Fig. 5. Cluster analysis of CA. (Left) The original scatter plots of $\Delta I/I_o$ versus $S.D.$ of events acquired with CA. Three independent trials **(a-c)** with identical measurement conditions were performed. Specifically, the measurements were performed with MspA-90PBA in a buffer of 1.5 M KCl, 100 mM MOPS, pH 7.0 and a +100 mV bias was continually applied. CA was added to *cis* with a final concentration of 1 mM. Results in each scatter plot were from a 20 min continuously recorded trace. **a)** 1076 events, **b)** 1075 events and **c)** 976 events were respectively included in each scatter plot. **(Right)** The scatter plots of $\Delta I/I_o$ versus $S.D.$ after cluster analysis treatment. To remove background noise events in the left scatter plots, the raw data were further treated by cluster analysis using DBSCAN. The epsilon was set to 0.08 and the min_samples was set to 11. Each right scatter plot demonstrates the same set of data however with the background noise events removed. After the treatment, **a)** 1013 events, **b)** 1000 events and **c)** 934 events were respectively retained. All statistical results are also demonstrated in **Supplementary Table 3**.

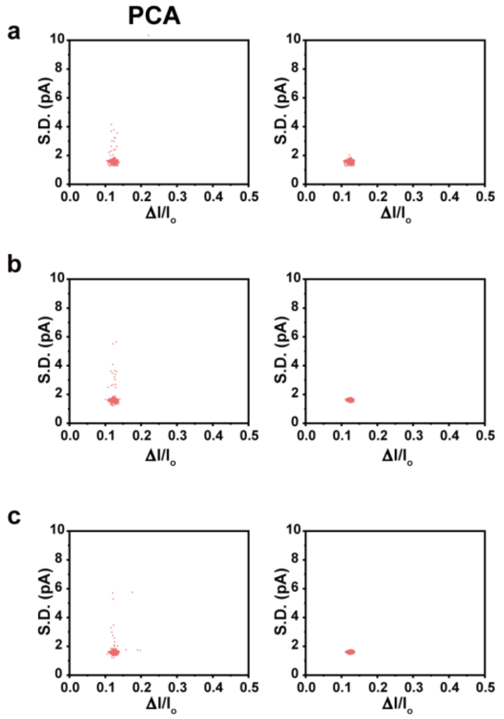

Supplementary Fig. 6. Cluster analysis of PCA. (Left) The original scatter plots of $\Delta I/I_o$ versus $S.D.$ of events acquired with PCA. Three independent trials **(a-c)** with identical measurements conditions were performed. Specifically, the measurements were performed with MspA-90PBA in a buffer of 1.5 M KCl, 100 mM MOPS, pH 7.0 and a +100 mV bias was continually applied. PCA was added to *cis* with a final concentration of 2 mM. Results in each scatter plot were from a 20 min continuously recorded trace. **a)** 904 events, **b)** 1074 events and **c)** 931 events were respectively included in each scatter plot. **(Right)** The scatter plots of $\Delta I/I_o$ versus $S.D.$ after cluster analysis treatment. To remove background noise events in the left scatter plots, the raw data were further treated by cluster analysis using DBSCAN. The epsilon was set to 0.08 and the min_samples was set to 11. Each right scatter plot demonstrates the same set of data however with the background noise events removed. After the treatment, **a)** 883 events, **b)** 1003 events and **c)** 868 events were respectively retained. All statistical results are also demonstrated in **Supplementary Table 3**.

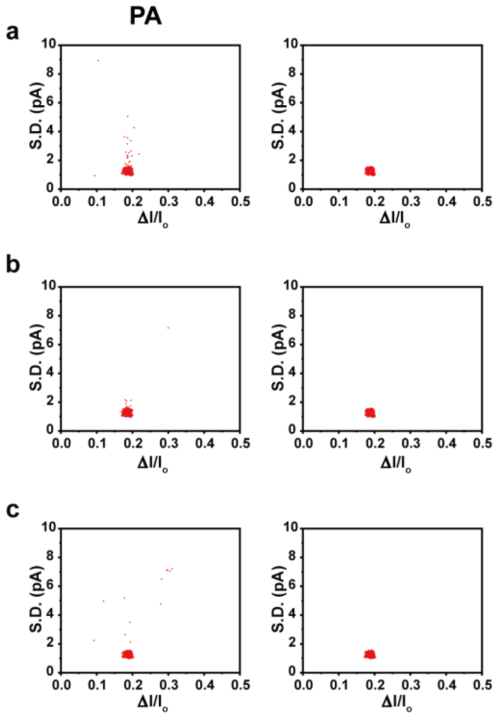

Supplementary Fig. 7. Cluster analysis of PA. (Left) The original scatter plots of $\Delta I/I_o$ versus $S.D.$ of events acquired with PA. Three independent trials **(a-c)** with identical measurement conditions were performed. Specifically, the measurements were performed with MspA-90PBA in a buffer of 1.5 M KCl, 100 mM MOPS, pH 7.0 and a +100 mV bias was continually applied. PA was added to *cis* with a final concentration of 0.5 mM. Results in each scatter plot were from a 20 min continuously recorded trace. **a)** 1497 events, **b)** 1516 events and **c)** 1193 events were respectively included in each scatter plot. **(Right)** The scatter plots of $\Delta I/I_o$ versus $S.D.$ after cluster analysis treatment. To remove background noise events in the left scatter plots, the raw data were further treated by cluster analysis using DBSCAN. The epsilon was set to 0.08 and the min_samples was set to 11. Each right scatter plot demonstrates the same set of data however with the background noise events removed. After the treatment, **a)** 1456 events, **b)** 1456 events and **c)** 1168 events were respectively retained. All statistical results are also demonstrated in **Supplementary Table 3**.

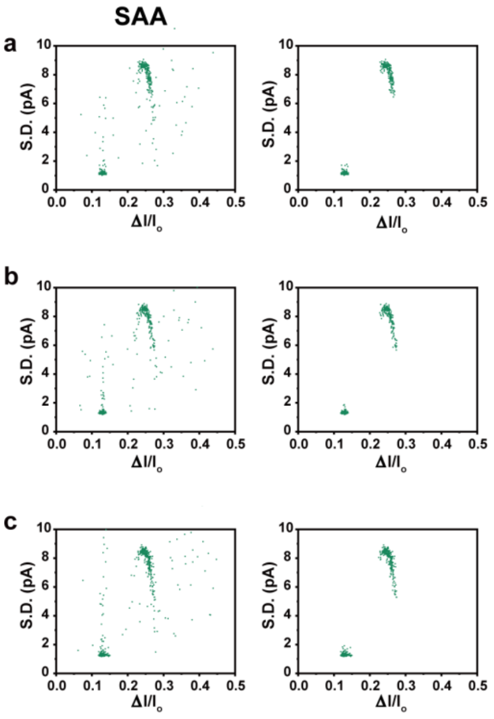

Supplementary Fig. 8. Cluster analysis of SAA. (Left) The original scatter plots of $\Delta I/I_o$ versus $S.D.$ of events acquired with SAA. Three independent trials **(a-c)** with identical measurement conditions were performed. Specifically, the measurements were performed with MspA-90PBA in a buffer of 1.5 M KCl, 100 mM MOPS, pH 7.0 and a +100 mV bias was continually applied. SAA was added to *cis* with a final concentration of 0.5 mM. Results in each scatter plot were from a 20 min continuously recorded trace. **a)** 622 events, **b)** 558 events and **c)** 639 events were respectively included in each scatter plot. **(Right)** The scatter plots of $\Delta I/I_o$ versus $S.D.$ after cluster analysis treatment. To remove background noise events in the left scatter plots, the raw data were further treated by cluster analysis using DBSCAN. The epsilon was set to 0.08 and the min_samples was set to 11. Each right scatter plot demonstrates the same set of data however with the background noise events removed. After the treatment, **a)** 522 events, **b)** 448 events and **c)** 517 events were respectively retained. All statistical results are also demonstrated in **Supplementary Table 3**.

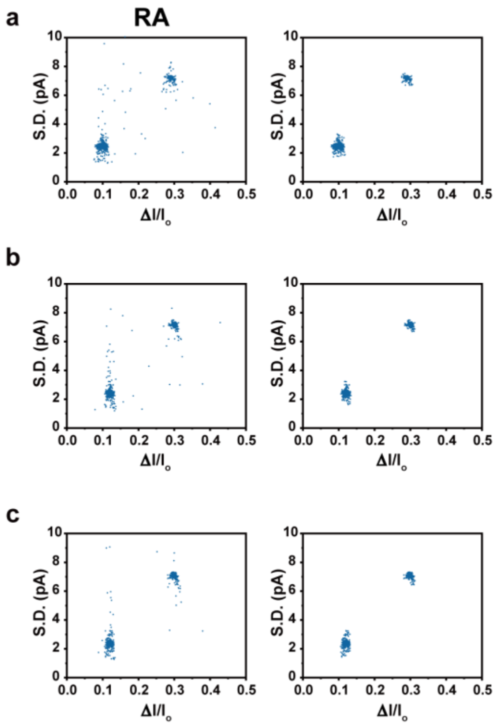

Supplementary Fig. 9. Cluster analysis of RA. (Left) The original scatter plots of $\Delta I/I_o$ versus $S.D.$ of events acquired with RA. Three independent trials **(a-c)** with identical measurement conditions were performed. Specifically, the measurements were performed with MspA-90PBA in a buffer of 1.5 M KCl, 100 mM MOPS, pH 7.0 and a +100 mV bias was continually applied. RA was added to *cis* with a final concentration of 0.3 mM. Results in each scatter plot were from a 20 min continuously recorded trace. **a)** 802 events, **b)** 756 events and **c)** 834 events were respectively included in each scatter plot. **(Right)** The scatter plots of $\Delta I/I_o$ versus $S.D.$ after cluster analysis treatment. To remove background noise events in the left scatter plots, the raw data were further treated by cluster analysis using DBSCAN. The epsilon was set to 0.08 and the min_samples was set to 11. Each right scatter plot demonstrates the same set of data however with the background noise events removed. After the treatment, **a)** 708 events, **b)** 683 events and **c)** 741 events were respectively retained. All statistical results are also demonstrated in **Supplementary Table 3**.

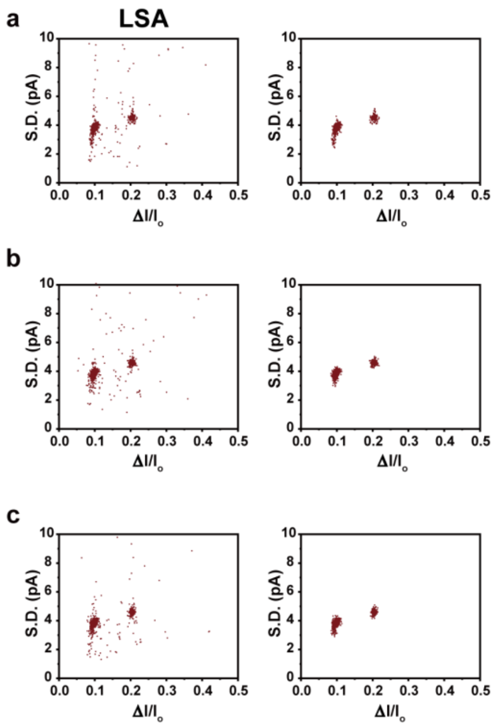

Supplementary Fig. 10. Cluster analysis of LSA. (Left) The original scatter plots of $\Delta I/I_o$ versus $S.D.$ of events acquired with LSA. Three independent trials **(a-c)** with identical measurement conditions were performed. Specifically, the measurements were performed with MspA-90PBA in a buffer of 1.5 M KCl, 100 mM MOPS, pH 7.0 and a +100 mV bias was continually applied. LSA was added to *cis* with a final concentration of 0.2 mM. Results in each scatter plot were from a 20 min continuously recorded trace. **a)** 882 events, **b)** 994 events and **c)** 992 events were respectively included in each scatter plot. **(Right)** The scatter plots of $\Delta I/I_o$ versus $S.D.$ after cluster analysis treatment. To remove background noise events in the left scatter plots, the raw data were further treated by cluster analysis using DBSCAN. The epsilon was set to 0.08 and the min_samples was set to 11. Each right scatter plot demonstrates the same set of data however with the background noise events removed. After the treatment, **a)** 769 events, **b)** 868 events and **c)** 873 events were respectively retained. All statistical results are also demonstrated in **Supplementary Table 3**.

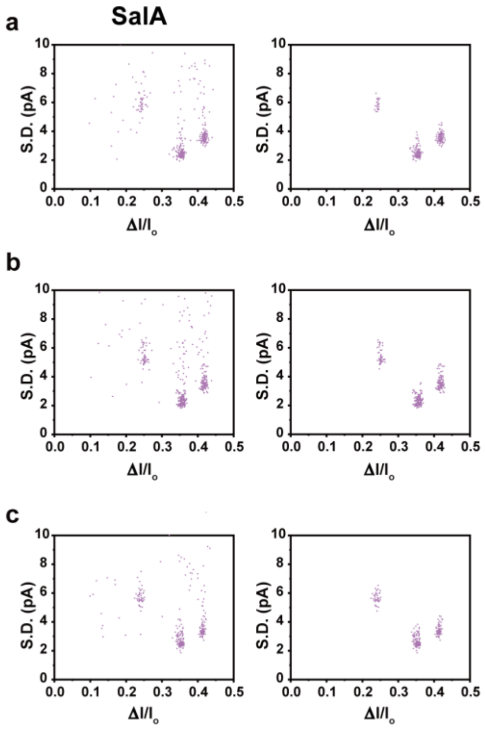

Supplementary Fig. 11. Cluster analysis of SalA. **(Left)** The original scatter plots of $\Delta I/I_o$ versus $S.D.$ of events acquired with SalA. Three independent trials **(a-c)** with identical measurement conditions were performed. Specifically, the measurements were performed with MspA-90PBA in a buffer of 1.5 M KCl, 100 mM MOPS, pH 7.0 and a +100 mV bias was continually applied. SalA was added to *cis* with a final concentration of 0.03 mM. Results in each scatter plot were from a 20 min continuously recorded trace. **a)** 360 events, **b)** 431 events and **c)** 329 events were respectively included in each scatter plot. **(Right)** The scatter plots of $\Delta I/I_o$ versus $S.D.$ after cluster analysis treatment. To remove background noise events in the left scatter plots, the raw data were further treated by cluster analysis using DBSCAN. The epsilon was set to 0.08 and the min_samples was set to 11. Each right scatter plot demonstrates the same set of data however with the background noise events removed. After the treatment, **a)** 247 events, **b)** 297 events and **c)** 240 events were respectively retained. All statistical results are also demonstrated in **Supplementary Table 3**.

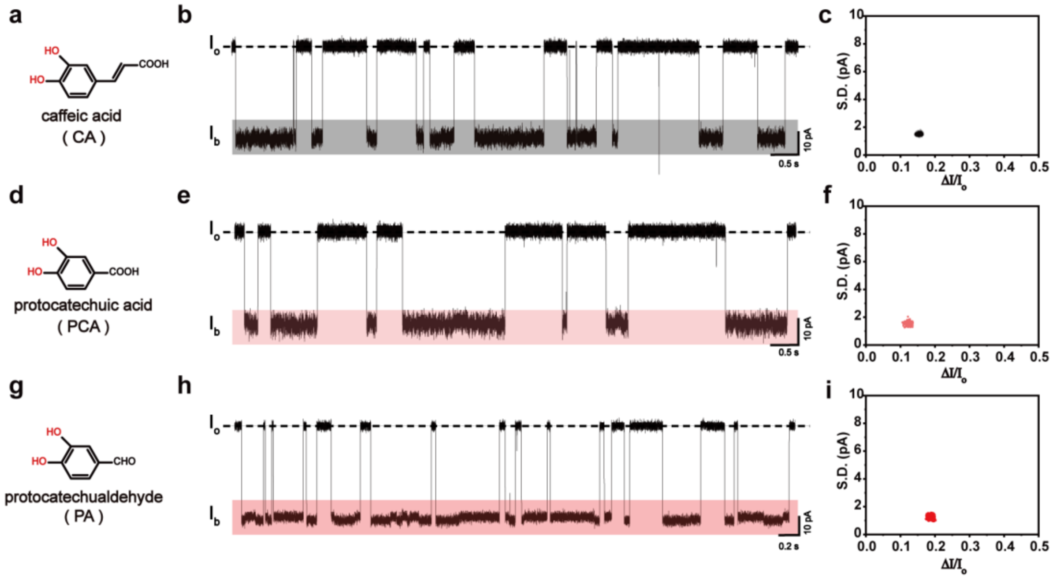

Supplementary Fig. 12. Nanopore sensing of salvianolic acids with a single 1, 2-dihydroxyl group. All measurements were carried out using MspA-90PBA in a buffer of 1.5 M KCl, 100 mM MOPS, pH 7.0 and a +100 mV bias was continually applied (**Methods**). The analytes, including **(a)** CA, **(d)** PCA and **(g)** PA were separately added to *cis* with a final concentration of 1 mM, 2 mM and 0.5 mM, respectively. **(b, e, h)** Representative traces acquired with **(a)** CA, **(d)** PCA and **(g)** PA as the sole analyte. The scatter plot of $\Delta I/I_o$ versus $S.D.$ generated by results of CA **(c)** (n = 1013), PCA **(f)** (n = 883) and PA **(i)** (n = 1456) sensing.

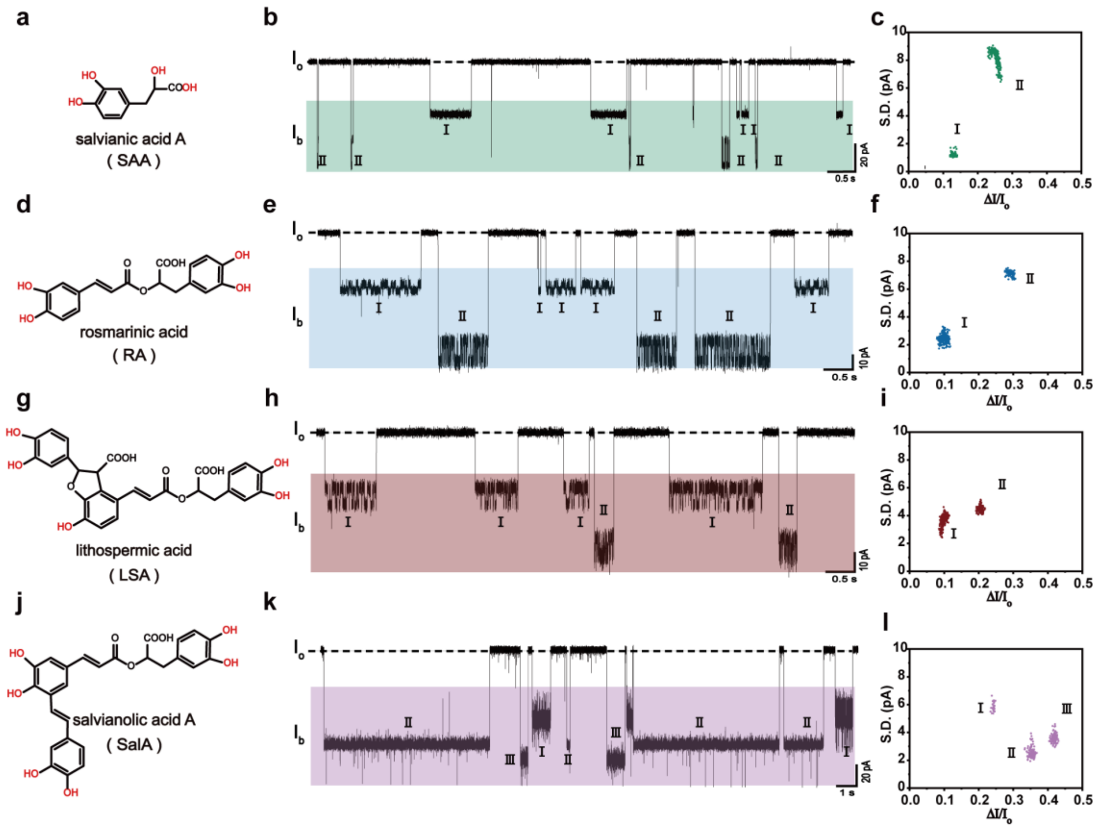

Supplementary Fig. 13. Nanopore sensing of salvianolic acids with multiple 1, 2-dihydroxyl groups. All measurements were carried out using MspA-90PBA in a buffer of 1.5 M KCl, 100 mM MOPS, pH 7.0 and a +100 mV bias was continually applied (**Methods**). The analytes, including **(a)** SAA, **(d)** RA, **(g)** LSA and **(j)** SalA were separately added to *cis* with a final concentration of 0.5 mM, 0.3 mM, 0.2 mM and 0.03 mM, respectively. **(b, e, h, k)** Representative traces acquired with **(b)** SAA, **(e)** RA, **(h)** LSA and **(k)** SalA as the sole analyte. The scatter plots of $\Delta I/I_o$ versus $S.D.$ generated by results of SAA **(c)** (n=522), RA **(f)** (n = 708), LSA **(i)** (n = 769) and SalA **(l)** (n = 247) sensing.

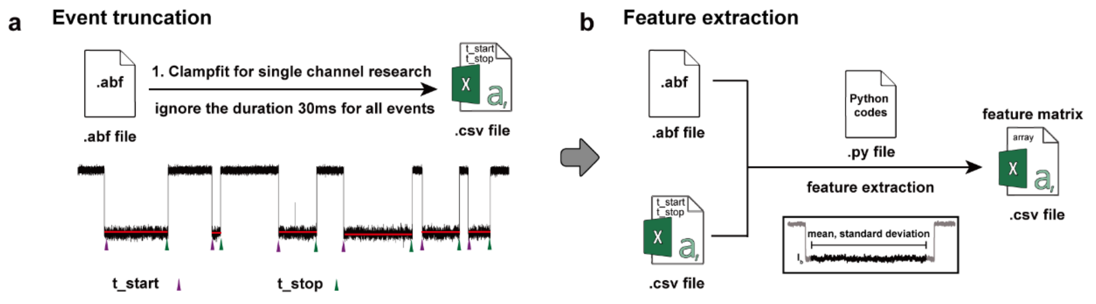

Supplementary Fig. 14. The workflow of event feature extraction. (a) Event segmentation. Events in a continually recorded trace were automatically detected by the "single channel research" function of Clampfit 10.7. The start time (t_start) and the end time (t_stop) of each event were recorded as a timestamp in a .csv file. Only events with a duration of more than 30 ms were collected for further analysis. **(b)** Feature extraction. The raw trace file and the corresponding timestamp file were simultaneously loaded to a custom Python program for event feature extraction (**Methods**). Two event features, including $\Delta I/I_o$ and $S.D.$ were extracted to form a feature matrix. The feature matrix results were saved as a .csv file for all subsequent machine learning operations.

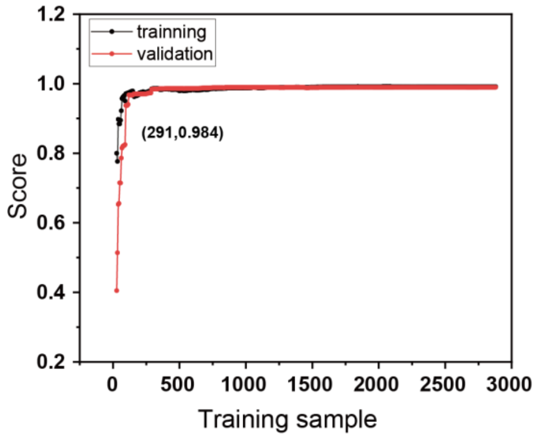

Supplementary Fig. 15. The learning curves. The training and the validation scores with varying amount of input training samples were respectively derived to form learning curves. A 10-fold cross validation was used. When the training sample exceeds 291, the validation score achieves 0.984. When the size of the input training sample increases, the training and the validation score start merging with each other, indicating that overfitting of the model was not happening.

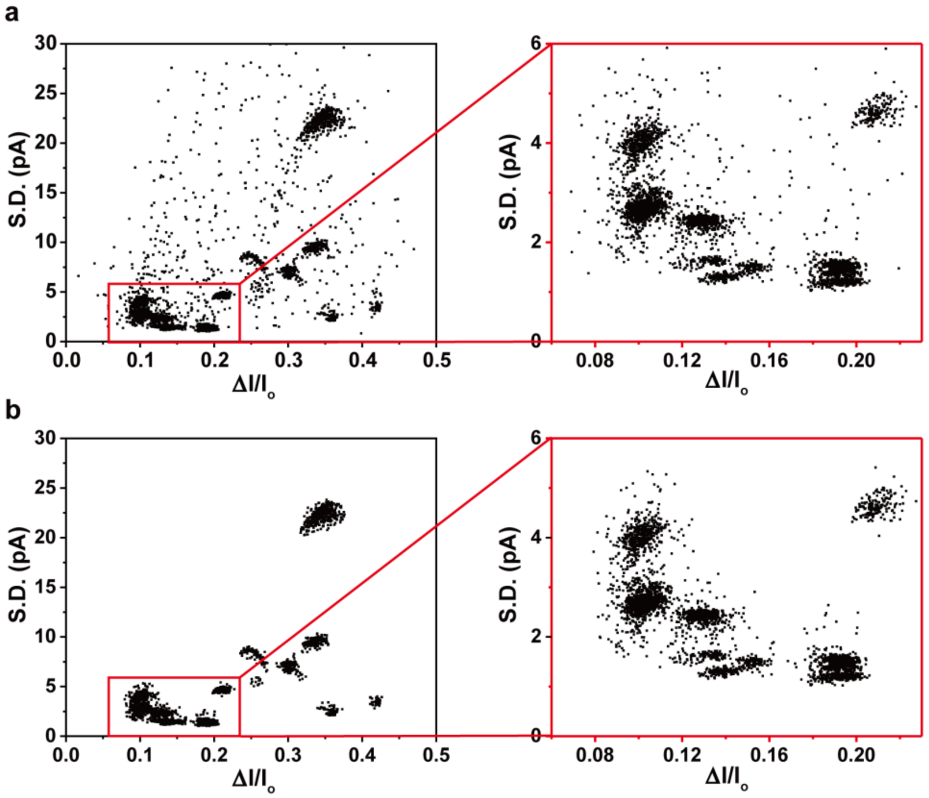

Supplementary Fig. 16. Cluster analysis of all eight analytes. (a) Left: The scatter plot of $\Delta I/I_o$ versus $S.D.$ of events acquired with eight analytes when simultaneously sensed by the same nanopore (n = 4589). All measurements were carried out using MspA-90PBA in a buffer of 1.5 M KCl, 100 mM MOPS, pH 7.0 (**Methods**). All analytes were added to *cis* to reach the desired final concentrations and a +100 mV bias was continually applied. Specially, the final concentration of CA and SAA was 40 µM, that of PCA was 100 µM, and that of PA, RA, LSA, SalA and SalB was 20 µM. Events in the scatter plot were from a 50 min continually recorded trace. **Right:** The zoomed-in view of the area marked with a red box in the left. **(b) Left:** The scatter plot of $\Delta I/I_o$ versus $S.D.$ after cluster analysis treatment. With DBSCAN algorithm, the background noise events in **(a)** were removed. The epsilon was set to 0.08 and the min_samples was set to 11. After treatment, 4366 events were retained. **Right:** The zoomed-in view of the area marked with a red box in the left.

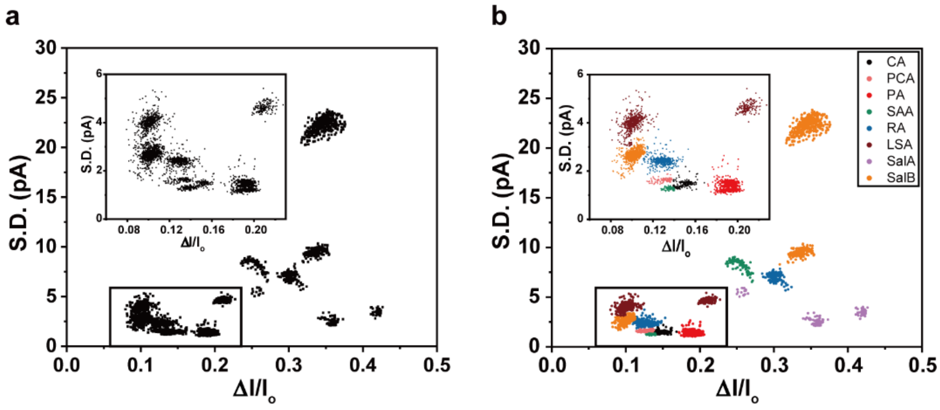

Supplementary Fig. 17. Prediction of events from eight analytes simultaneous sensing. (a) The scatter plot of $\Delta I/I_o$ versus $S.D.$ of events after cluster analysis treatment. All background noise events were removed with DBSCAN cluster analysis as described in **Supplementary Fig. 16**. Events in the scatter plot were from a 50 min continually recorded trace. **(b)** The scatter plot of $\Delta I/I_o$ versus $S.D.$ of result from model prediction (n = 4268). All events were labelled by the previously trained KNN model and color labelled in **b)**. A +100 mV bias was continually applied and all analytes were added in *cis* with a buffer of 1.5 M KCl, 100 mM MOPS, pH 7.0.

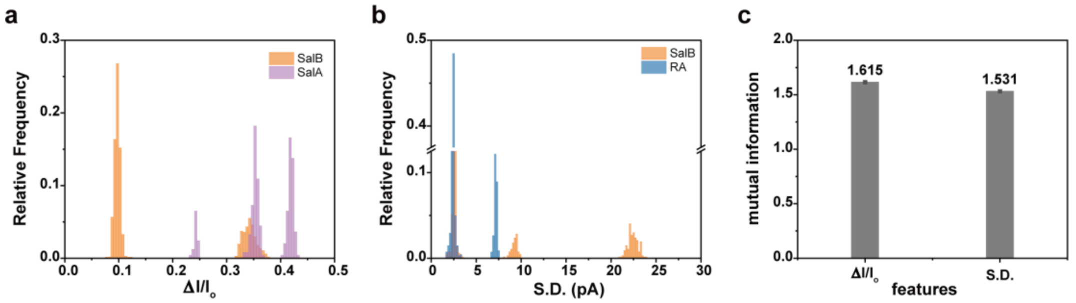

Supplementary Fig. 18. The comparison of mutual information for salvianolic acid identification. **(a)** The superimposed histogram of $\Delta I/I_o$ of events acquired with SalB and SalA. **(b)** The superimposed histogram of $S.D.$ of events acquired with SalB and RA. Obviously, event overlaps are clearly seen when only a single event parameter is used. **(c)** The mutual information values of $\Delta I/I_o$ and $S.D.$ derived from salvianolic acid identification. Mutual information, based on the concept of information theory, is used to measure the degree of interdependence between two random variables[1]. During machine learning, the mutual information can also be employed to evaluate the correlation between each feature and the target event labels[2]. The most informative features are then selected and considered in the subsequent model building process. Here, the mutual information values of $\Delta I/I_o$ and $S.D.$ were implemented using a Python based custom code. The core calculation code is a function of '$\mathrm{mutual\_info} = \mathrm{mutual\_info\_classif}\,(\mathrm{X}, \mathrm{Y})$' from the sklearn library, where the X stands for the features matrix of $\Delta I/I_o$ and $S.D.$, the Y stands for the event labels and the mutual_info refers to the mutual information values of the two features.

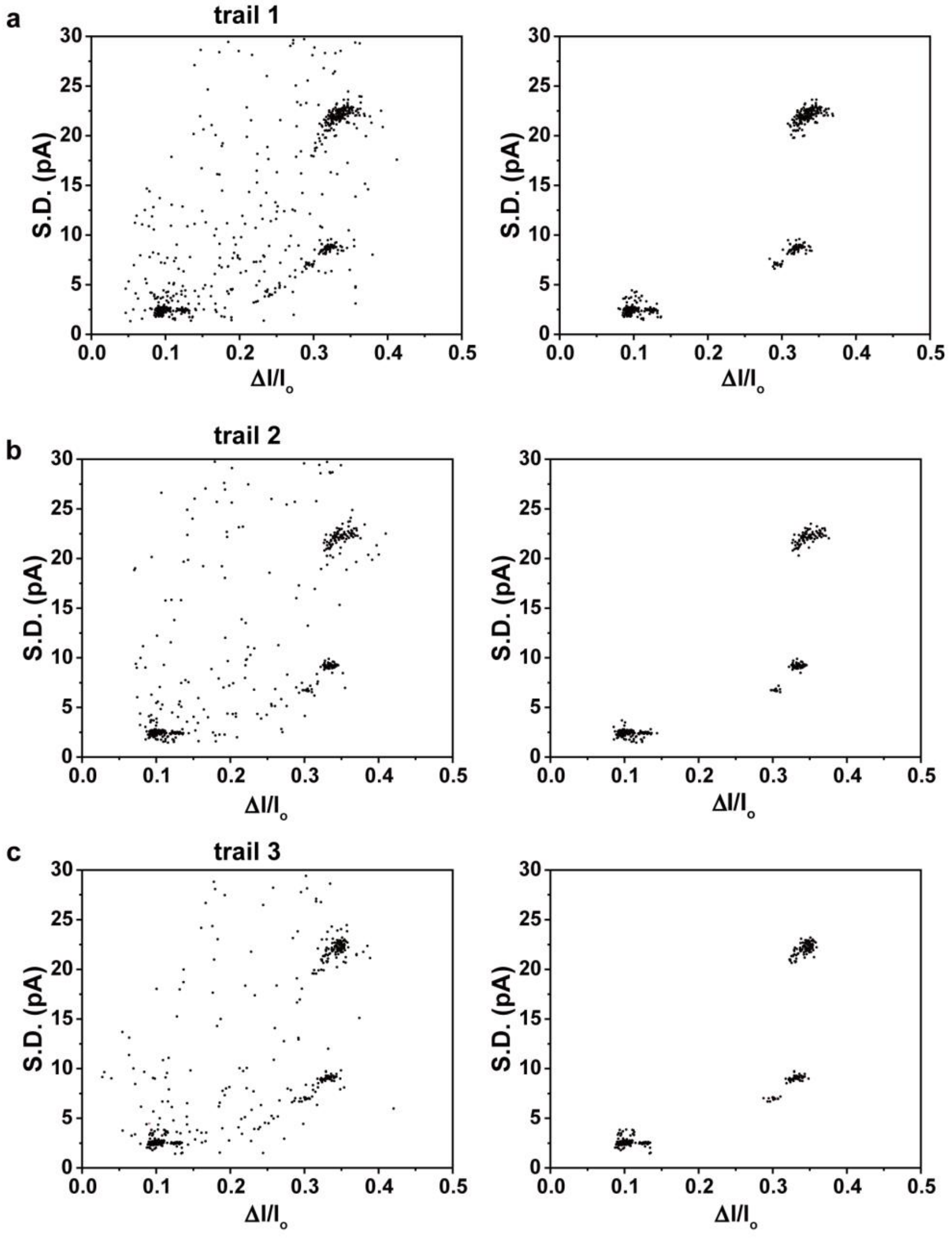

Supplementary Fig. 19. Cluster analysis of salvianolate injection. (Left) The scatter plots of $\Delta I/I_o$ versus $S.D.$ of events acquired with salvianolate injection. Three independent trials **(a-c)** with identical measurement conditions were performed. Specifically, the measurements were performed with MspA-90PBA in a buffer of 1.5 M KCl, 100 mM MOPS, pH 7.0 and a +100 mV bias was continually applied. The injection was added to *cis* with a final concentration of 0.04 mg/mL. Results in each scatter plot were from a 30 min continuously recorded trace. **a)** 1096 events, **b)** 807 events and **c)** 1199 events were respectively included in each scatter plot. **(Right)** The scatter plots of $\Delta I/I_o$ versus $S.D.$ after cluster analysis treatment. To remove background noise events in the left scatter plots, the raw data were further treated by cluster analysis using DBSCAN. The epsilon was set to 0.08 and the min_samples was set to 11. Each right scatter plot demonstrates the same set of data however with the background

noise events removed. After the treatment, **a)** 868 events, **b)** 631 events and **c)** 938 events were respectively retained.

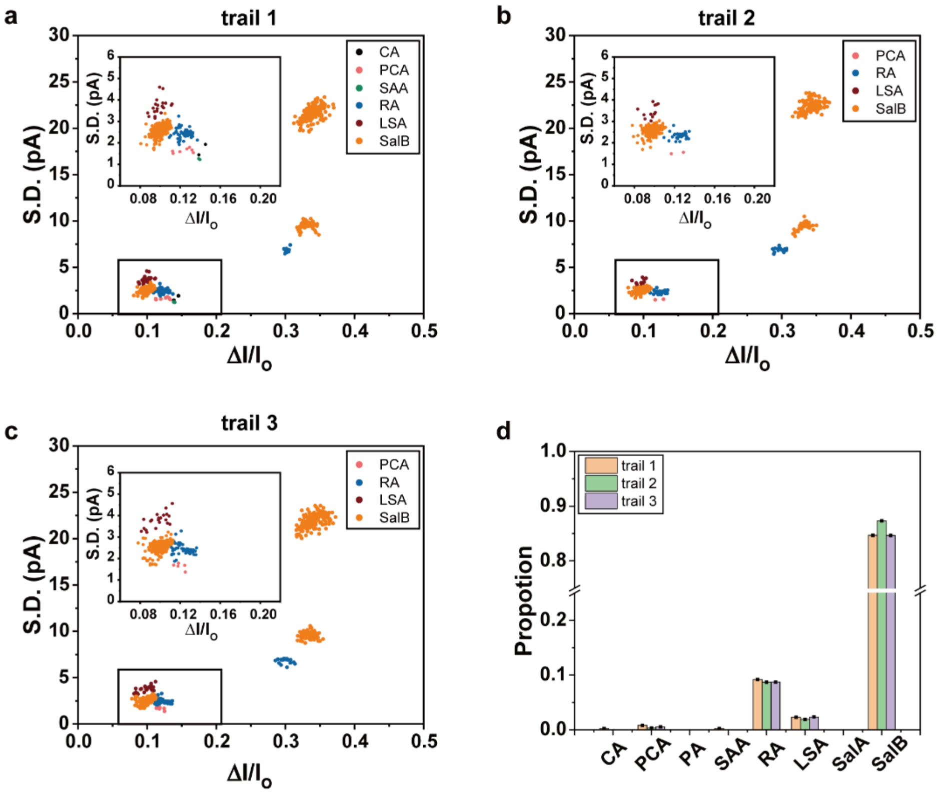

Supplementary Fig. 20. Results of salvianolate injection sensing. (a-c) The scatter plots of $\Delta I/I_o$ versus $S.D.$ of results acquired with salvianolate injection. All events were labelled with the previously trained KNN model. All noise background events were removed by DBSCAN clustering analysis (**Supplementary Fig. 19**). Events in each scatter plot were from a 30 min continually recorded trace and **a)** 846 events, **b)** 620 events and **c)** 903 events were respectively demonstrated in each scatter plot. **(d)** A demonstration of identified salvianolic acid events from the salvianolate injection. Results of three independent trials were simultaneously presented to show the consistency of the measurements. All measurements were performed with MspA-90PBA in a buffer of 1.5 M KCl, 100 mM MOPS, pH 7.0 and a +100 mV bias was continually applied. Salvianolate injection solution was added to *cis* with a final concentration of 0.04 mg/mL.

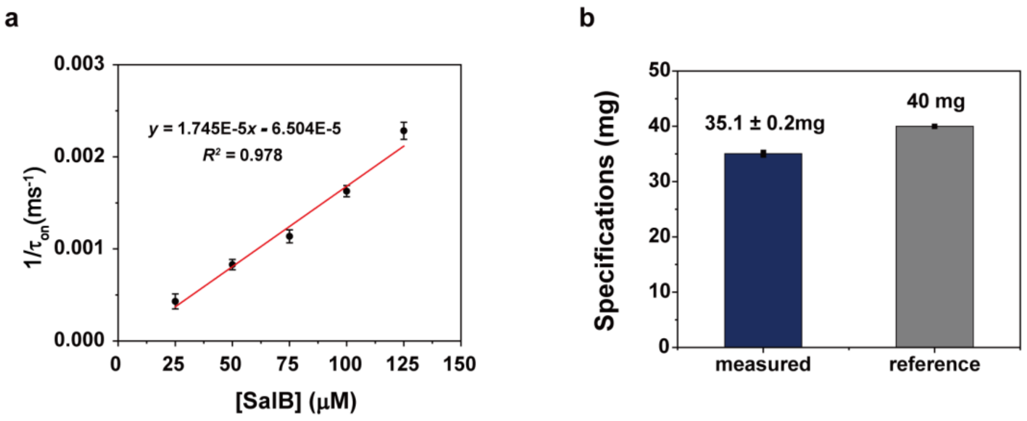

Supplementary Fig. 21. Quantitative analysis of salvianolate injection. (a) The calibration curve of SalB. All statistical data in this plot were acquired with different concentrations of SalB (**Supplementary Fig. 3**). The $1/\tau_{on}$ is linearly correlated with the SalB concentration and a coefficient factor (R^2) was shown. **(b)** The comparison of the measured and the reference values of magnesium salt of SalB. The measured values were derived as described in **Methods** and **Supplementary Fig. 20**. The reference value was derived from the product manual. Data in **(b)** are mean ± standard deviation values of results from independent measurements (N=3). The error bars represent standard deviation values. All statistical results are also demonstrated in **Supplementary Tables 1** and **4**. According to the results, the measured value is generally consistent with product manual.

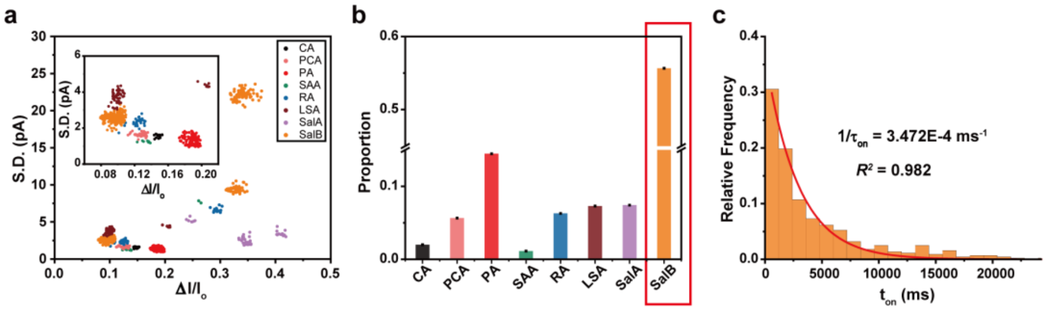

Supplementary Fig. 22. Quantitative analysis of eight salvianolic acids simultaneous sensing. **(a)** The event scatter plot of $\Delta I/I_o$ versus $S.D.$ of results acquired with a mixture of eight salvianolic acids. A total of 794 events were included (n = 794) and all event identities were predicted by machine learning. All analytes were added to *cis* to reach the desired final concentrations and a + 100 mV bias was continually applied. Specifically, the final concentration of CA and SAA was 10 µM, that of PCA was 20 µM, that of PA, RA, LSA and SalA was 5 µM and that of SalB was 25 µM. Events in the scatter plot were from a 40 min continually recorded trace. **(b)** A quantitative demonstration of identified salvianolic acid events from the mixture. **(c)** The histogram of t_{on} acquired from SalB events. By single-exponential fitting as described in **Supplementary Fig. 2**, the values of $1/\tau_{on}$ of SalB measured in the mixture was obtained and a coefficient factor (R^2) was shown. Subsequently, the concentration of SalB in the mixture can be derived as described in **Methods** and **Supplementary Fig. 21**.

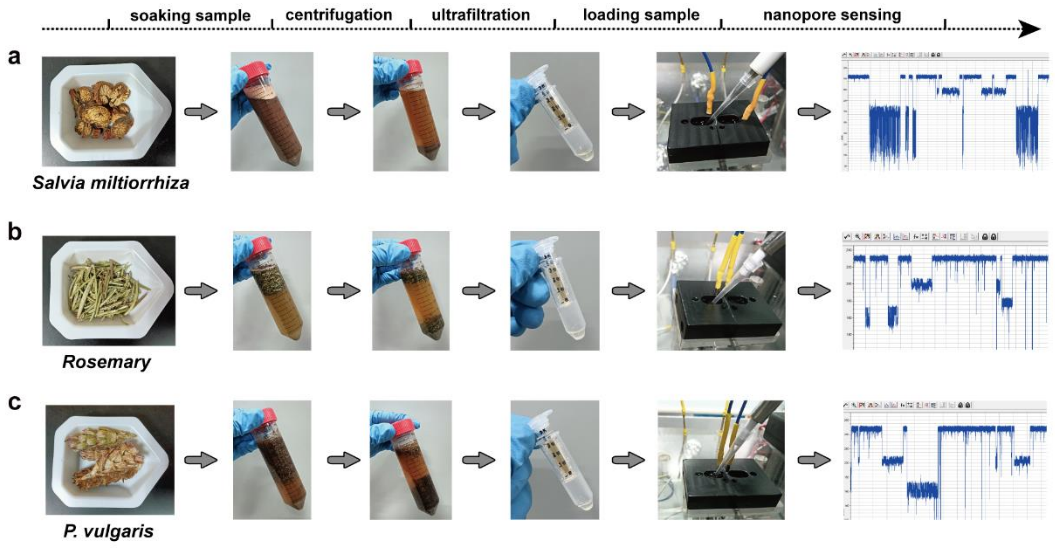

Supplementary Fig. 23. The workflow of nanopore herb analysis. (a) *Salvia miltiorrhiza*, **(b)** *Rosemary* and **(c)** *P. vulgaris* were separately crushed and soaked in Mili-Q water for 12 hours at 4 ℃. Then, the soaking liquid of each herb was collected and centrifuged at 4 ℃ and 1500 g for 10 minutes to collect the supernatant. Afterwards, the collected supernatant was further treated in a 3 kDa ultrafiltration tube at 4 ℃ and 1900 g for 30 minutes and the filtrate was collected for nanopore measurements. All nanopore measurements were carried out using MspA-90PBA in a buffer of 1.5 M KCl, 100 mM MOPS, pH 7.0 and a +100 mV bias was continually applied. To initial the measurement, 20 µL filtrate from each type of herb was added to *cis* and the corresponding nanopore events were immediately observed.

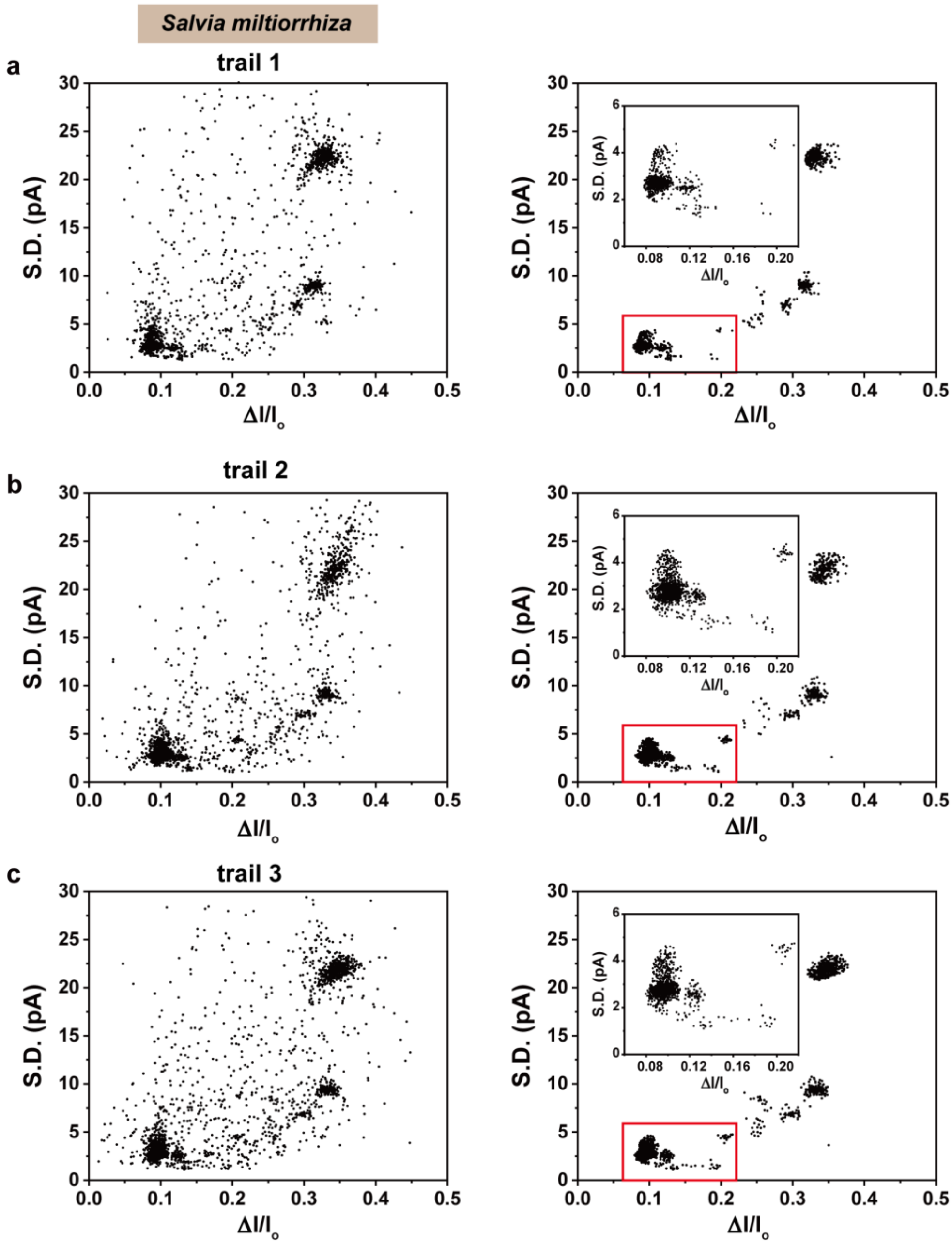

Supplementary Fig. 24. Outlier analysis of events acquired with *Salvia miltiorrhiza* using One-Class SVM. **(a-c) Left:** the event scatter plots of $\Delta I/I_o$ versus $S.D.$ of events extracted from 80 min continually recorded traces acquired using *Salvia miltiorrhiza*. **a)** 2315, **b)** 2421 and **c)** 2910 events respectively acquired with *Salvia miltiorrhiza* in different trials were demonstrated. **Right:** the corresponding event scatter plots of $\Delta I/I_o$ versus $S.D.$ however treated with outlier analysis using One-Class SVM. The retained events, which were also recognized as inlier events were identified according to the training data previously acquired with standard salvianolic acids analytes. **a)** 1490, **b)** 1715 and **c)** 2061 inlier events were retained in each corresponding scatter plot. The parameter 'nu' of One-Class SVM was set 0.01.

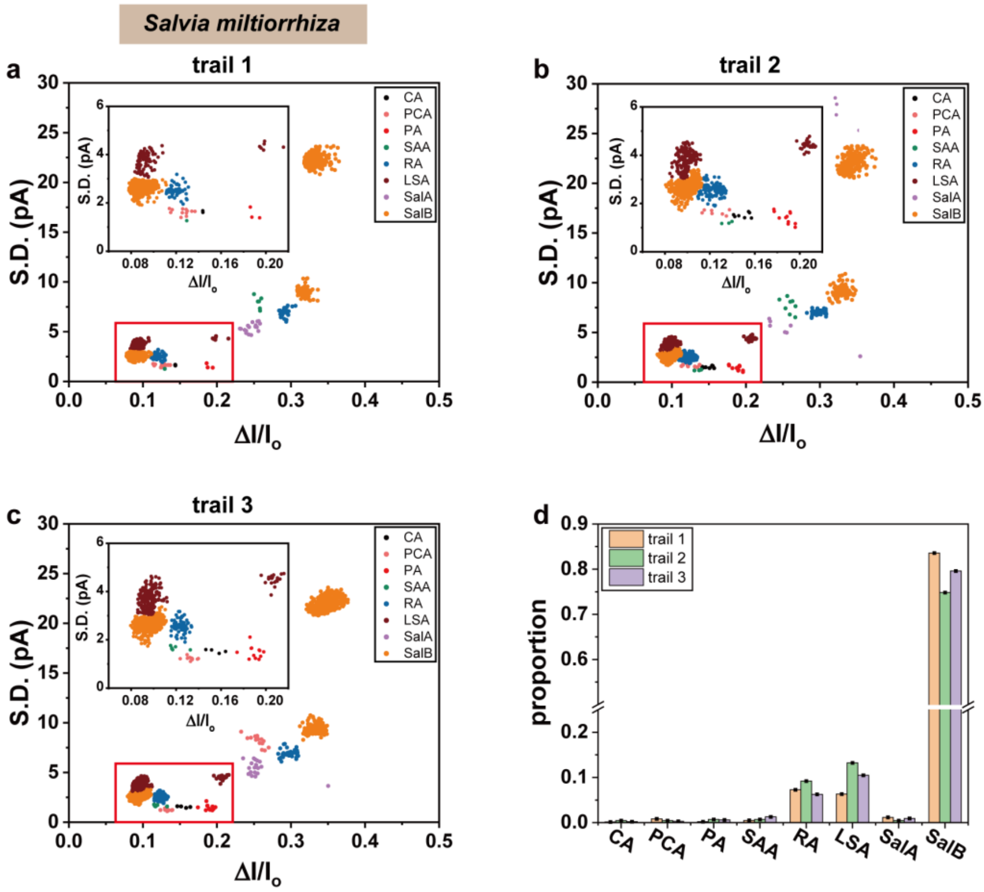

Supplementary Fig. 25. ***Salvia miltiorrhiza*** **analysis. (a-c)** The event scatter plots of $\Delta I/I_o$ versus $S.D.$ generated by events acquired with *Salvia miltiorrhiza*. All events were identified and labelled by the previously trained KNN model. All interference events were removed by One-Class SVM outlier analysis (**Supplementary Fig. 24**). Events in each scatter plot were from 80 min continually recorded traces respectively acquired in different trials. **a)** 1456, **b)** 1585 and **c)** 1969 events were respectively demonstrated in each scatter plot. **(d)** A histogram plot of identified salvianolic acid events from *Salvia miltiorrhiza*. Results of three independent trials were simultaneously shown to show the result consistency. All measurements were performed with MspA-90PBA in a buffer of 1.5 M KCl, 100 mM MOPS, pH 7.0. A +100 mV bias was continually applied. To initiate the measurement, 20 µL filtrate extracted from *Salvia miltiorrhiza* was added to *cis*.

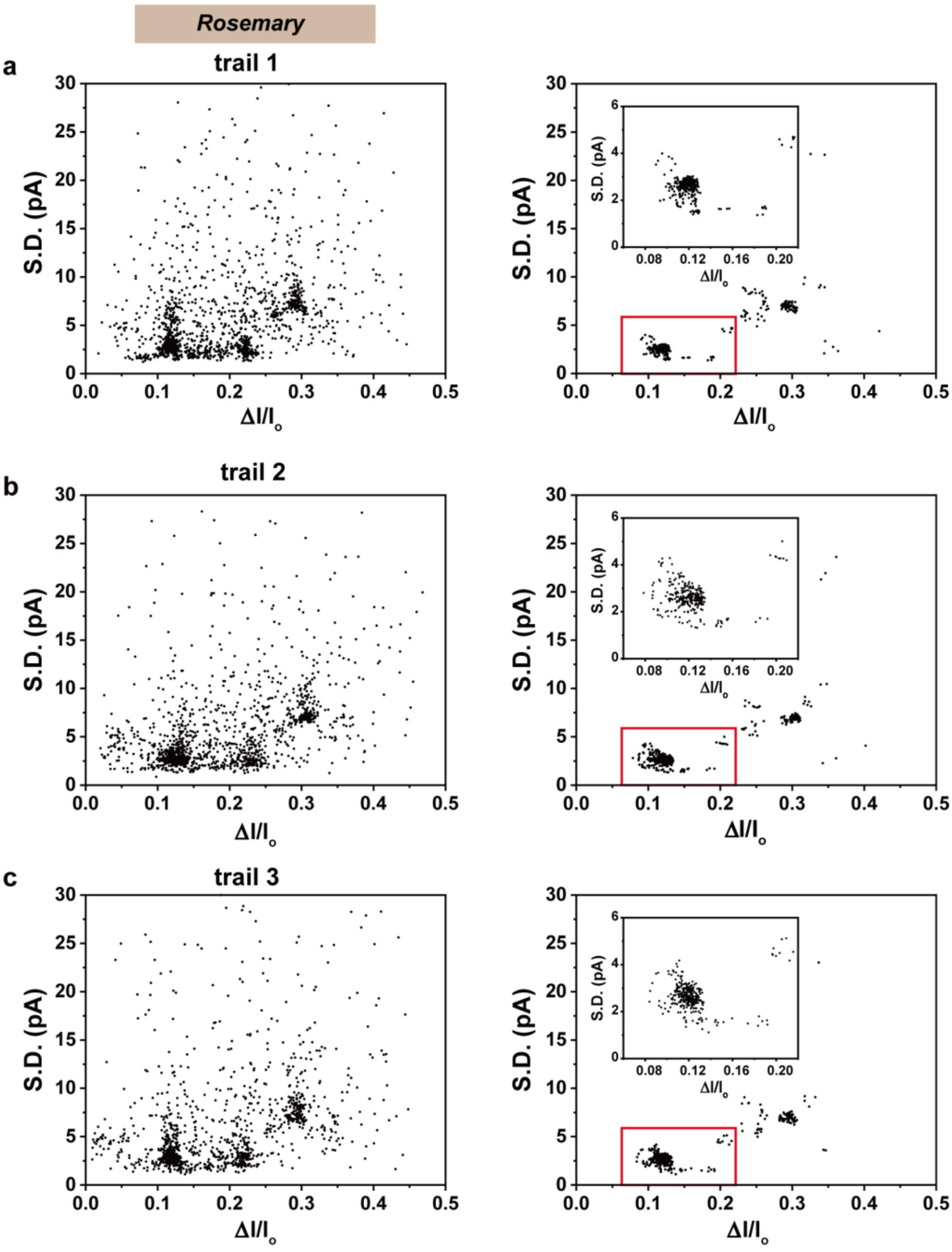

Supplementary Fig. 26. Outlier analysis of events acquired with *Rosemary* using One-Class SVM. **(a-c) Left:** the event scatter plots of $\Delta I/I_o$ versus $S.D.$ of events extracted from 80 min continually recorded traces acquired using *Rosemary*. **a)** 1629, **b)** 1376 and **c)** 1269 events respectively acquired with *Rosemary* in different trials were demonstrated. **Right:** the corresponding event scatter plots of $\Delta I/I_o$ versus $S.D.$ however treated with outlier analysis using One-Class SVM. The retained events, which were also recognized as inlier events were identified according to the training data previously acquired with standard salvianolic acids analytes. **a)** 440, **b)** 377 and **c)** 432 inlier events were retained in each corresponding scatter plot. The parameter 'nu' of One-Class SVM was set 0.01.

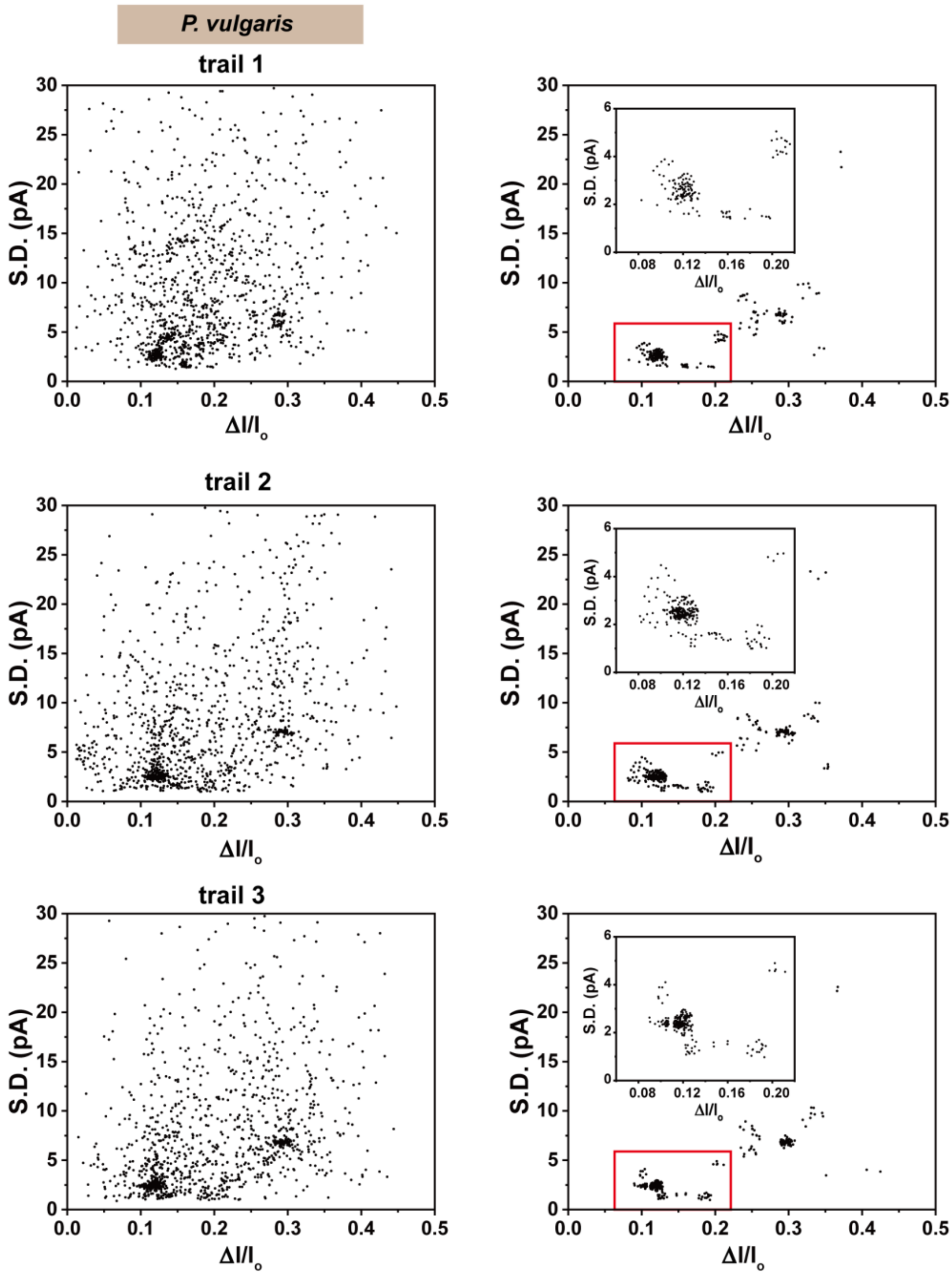

Supplementary Fig. 27. Outlier analysis of events acquired with *P. vulgaris* using One-Class SVM. **(a-c) Left:** the event scatter plots of $\Delta I/I_o$ versus $S.D.$ of events extracted from 80 min continually recorded traces acquired using *P. vulgaris*. **a)** 1656, **b)** 1266 and **c)** 1339 events respectively acquired with *P. vulgaris* in different trails were demonstrated. **Right:** the corresponding event scatter plots of $\Delta I/I_o$ versus $S.D.$ however treated with outlier analysis using One-Class SVM. The retained events, which were also recognized as inlier events were identified according to the training data previously acquired with standard salvianolic acids analytes. **a)** 253, **b)** 333 and **c)** 338 inlier events were retained in each corresponding scatter plot. The parameter 'nu' of One-Class SVM was set 0.01.

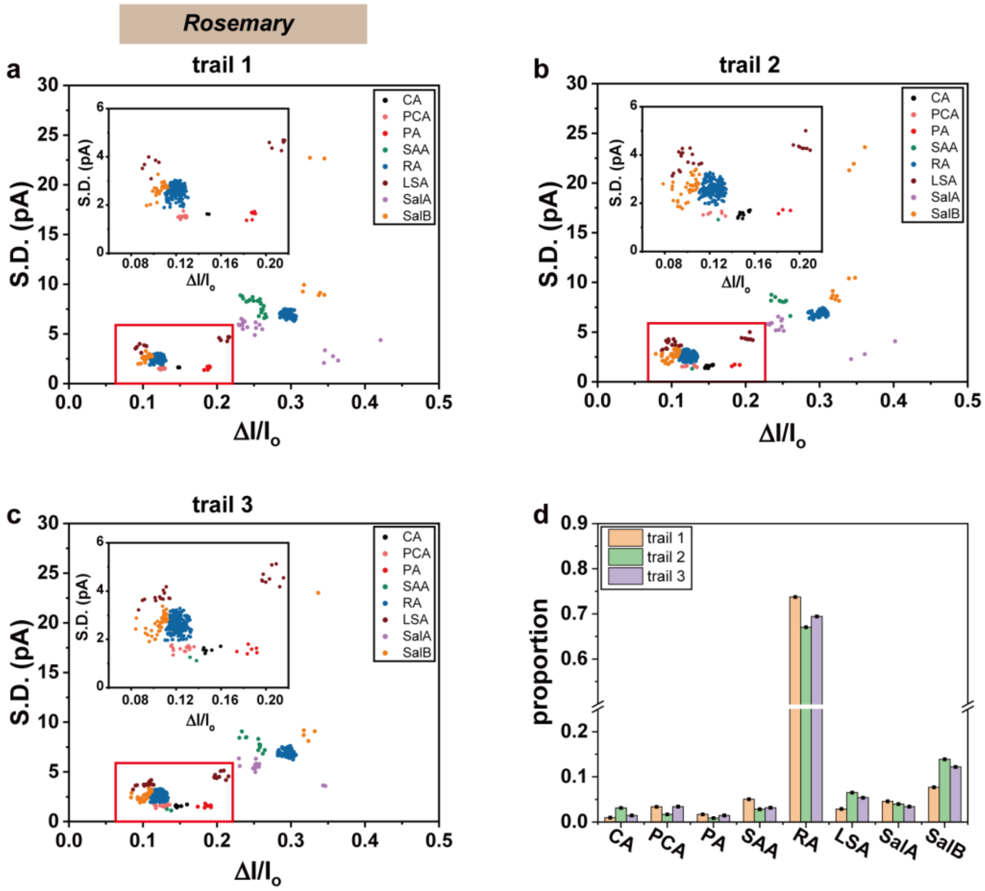

Supplementary Fig. 28. ***Rosemary*** **analysis. (a-c)** The event scatter plots of $\Delta I/I_o$ versus $S.D.$ generated by events acquired with *Rosemary*. All events were identified and labelled by the previously trained KNN model. All interference events were removed by One-Class SVM outlier analysis (**Supplementary Fig. 26**). Events in each scatter plot were from 80 min continually recorded traces respectively acquired in different trials. **a)** 415, **b)** 352 and **c)** 409 events were respectively demonstrated in each scatter plot. **(d)** A histogram plot of identified salvianolic acid events from *Rosemary*. Results of three independent trials were simultaneously shown to show the result consistency. All measurements were performed with MspA-90PBA in a buffer of 1.5 M KCl, 100 mM MOPS, pH 7.0. A +100 mV bias was continually applied. To initiate the measurement, 20 µL filtrate extracted from *Rosemary* was added to *cis*.

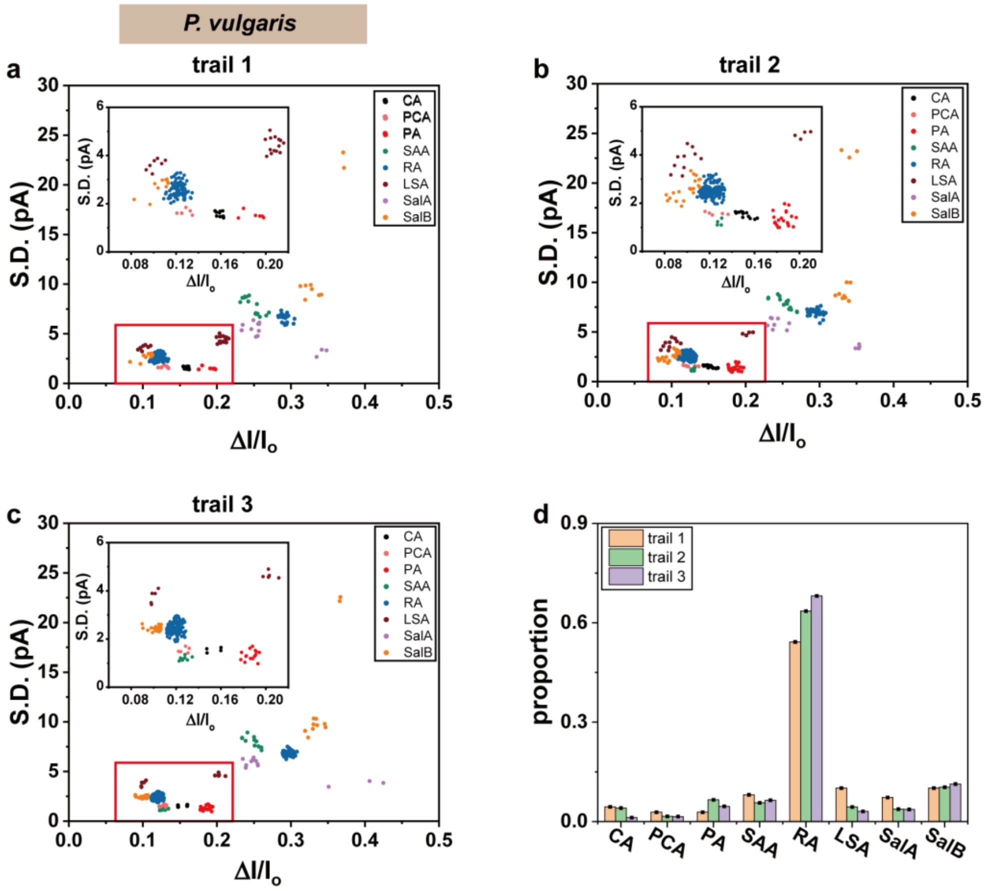

Supplementary Fig. 29. ***P. vulgaris*** **analysis. (a-c)** The event scatter plots of $\Delta I/I_o$ versus $S.D.$ generated by events acquired with *P. vulgaris*. All events were identified and labelled by the previously trained KNN model. All interference events were removed by One-Class SVM outlier analysis (**Supplementary Fig. 27**). Events in each scatter plot were from 80 min continually recorded traces respectively acquired in different trials. **a)** 247, **b)** 318 and **c)** 326 events were respectively demonstrated in each scatter plot. **(d)** A histogram plot of identified salvianolic acid events from *P. vulgaris*. Results of three independent trials were simultaneously shown to show the result consistency. All measurements were performed with MspA-90PBA in a buffer of 1.5 M KCl, 100 mM MOPS, pH 7.0. A +100 mV bias was continually applied. To initiate the measurement, 20 μL filtrate extracted from *P. vulgaris* was added to *cis*.

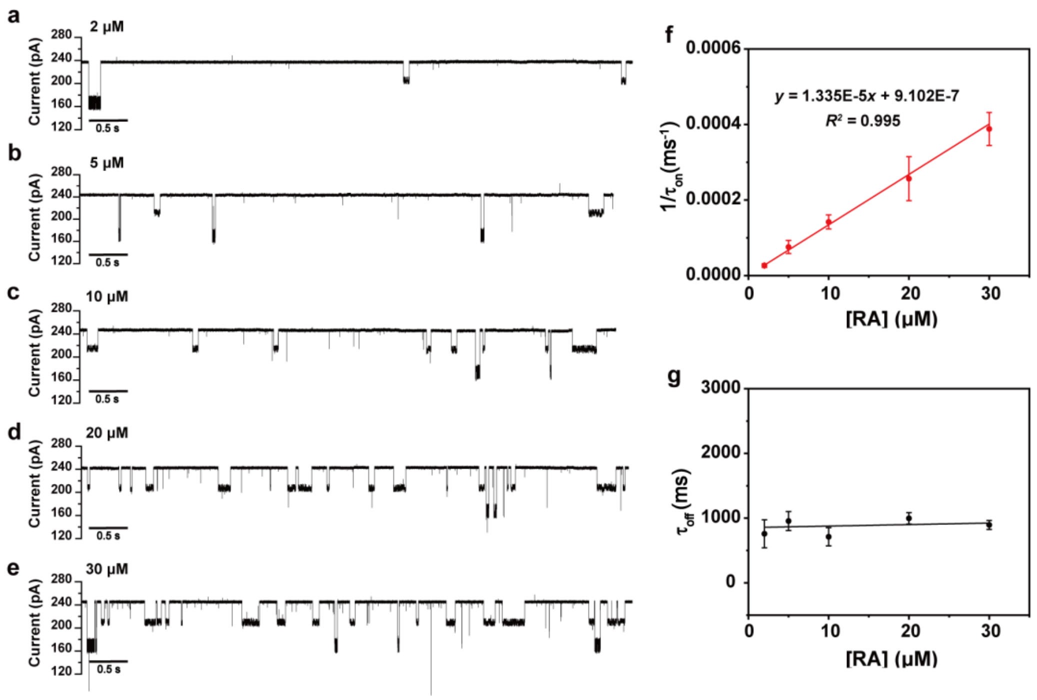

Supplementary Fig. 30. The concentration dependence of RA sensing. (a-e) Representative traces of RA sensing performed with different RA concentrations. All measurements were performed using MspA-90PBA in a buffer of 1.5 M KCl, 100 mM MOPS, pH 7.0 and a +100 mV bias was continually applied (**Methods**). RA was added to *cis* with a final concentration of 2-30 μM. **(f)** The calibration curve of RA. The plot of $1/\tau_{on}$ versus the RA concentration was demonstrated. The $1/\tau_{on}$ is linearly correlated with the RA concentration. **(g)** The plot of τ_{off} versus the RA concentration. The τ_{off} remains constant despite of the change of the RA concentration. The data in **(f-g)** show mean ± standard deviation values derived from results of three independent measurements (N=3). The error bars represent standard deviation values. All statistic results are also demonstrated in **Supplementary Table 7**.

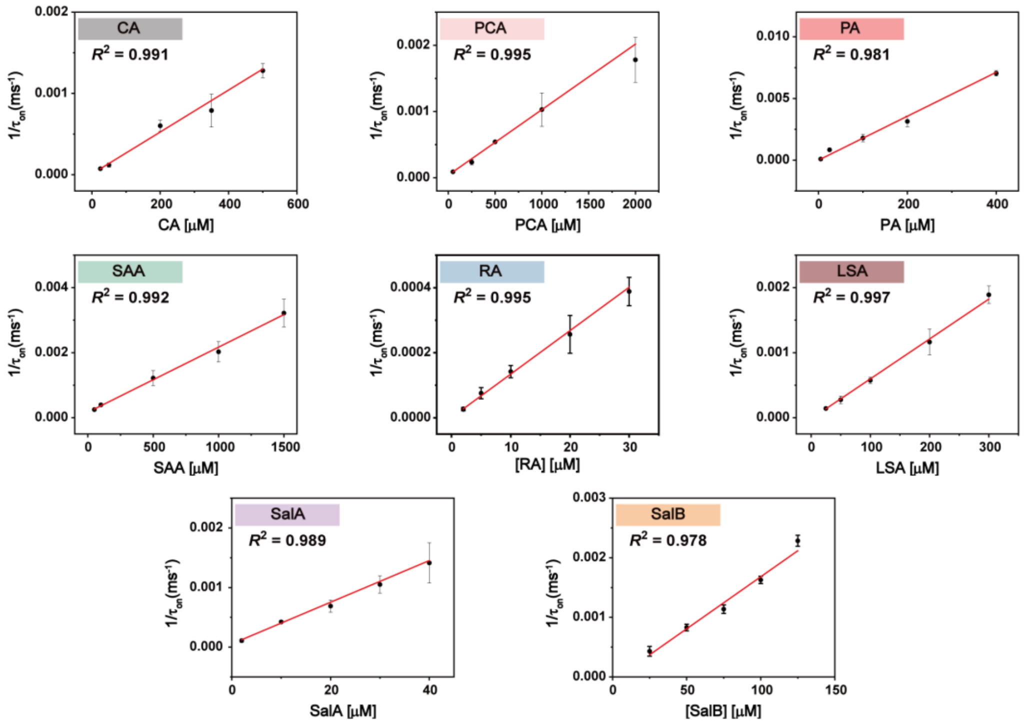

Supplementary Fig. 31. The range of effective concentration. The calibration curves of eight salvianolic acids within the ranges of their effective concentration. For each analyte, the $1/\tau_{on}$ is linearly correlated with the analyte concentration and a coefficient factor (R^2) was also shown. The data show mean ± standard deviation values derived from results of three independent measurements (N=3). The error bars represent standard deviation values. The range of effective concentrations for different salvianolic acid analytes are also summarized in **Supplementary Table 8**.

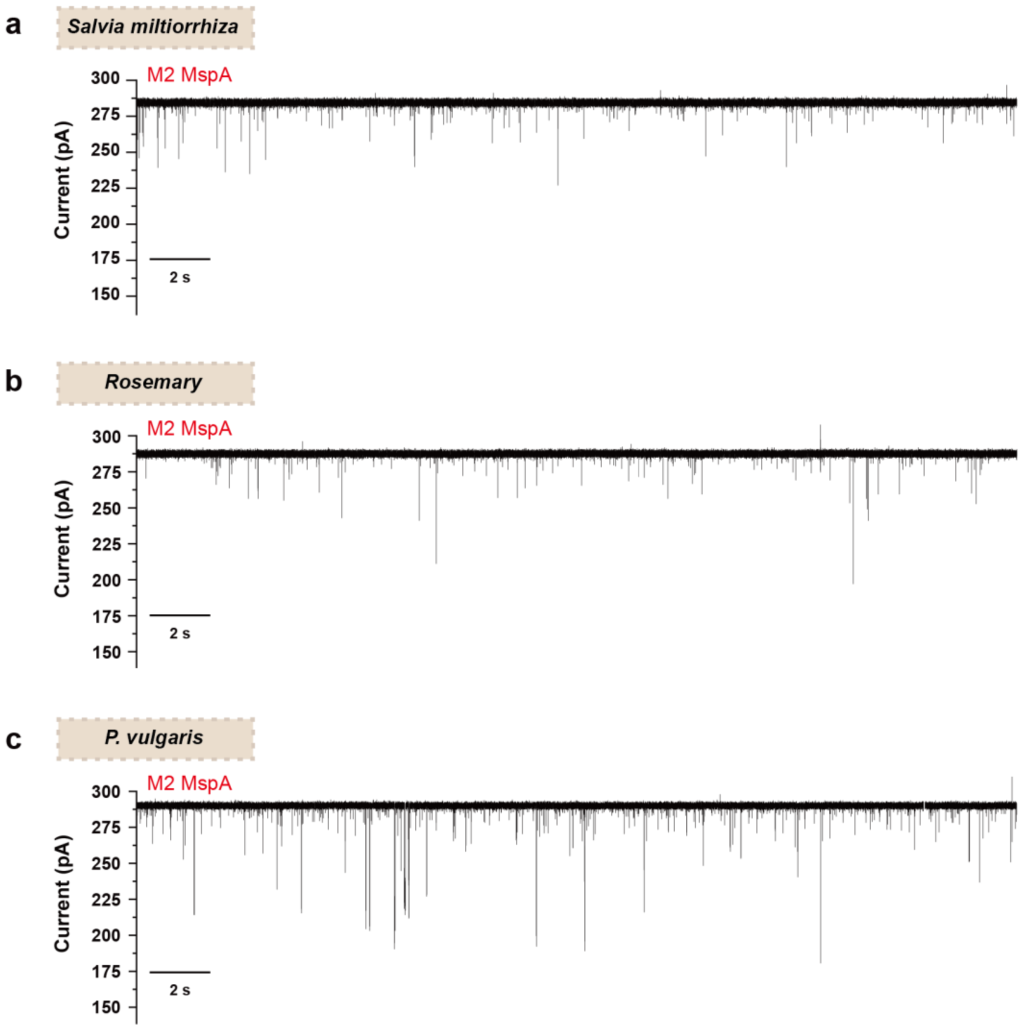

Supplementary Fig. 32. Herb analysis by M2 MspA. All measurements were carried out using M2 MspA in a buffer of 1.5 M KCl, 100 mM MOPS, pH 7.0 and a +100 mV was continually applied. 20 μL filtrate of natural herbs were respectively added to *cis*. **(a-c)** Representative traces acquired from nanopore analysis of **(a)** *Salvia miltiorrhiza*, **(b)** *Rosemary* and **(c)** *P. vulgaris* using M2 MspA. No events with well-defined event features were consistently observed. This is expected because the M2 MspA, which lacks a PBA adapter, fails to reversibly react with any *cis*-diol components in natural herb samples. Events caused by analyte collision to the pore lumen would not provide any well-defined sensing information for further analysis. These results again confirmed that the appended PBA adapter and the hetero-octamer assembly is critical in the gaining of the herb analysis function.

References

1. Cover, T. M. *Elements of information theory*. (John Wiley & Sons, 1999).
2. Battiti, R. Using mutual information for selecting features in supervised neural net learning. *IEEE Transactions on Neural Networks* **5**, 537-550 (1994).
