# Supplementary material for: Nanopore analysis of salvianolic acids in herbal medicines
Source: Nat Commun. 2024 Mar 5;15:1970. doi: 10.1038/s41467-024-45543-1 (PMC10915175; doi:10.1038/s41467-024-45543-1)
Supplement: Supplementary file 3 — Description of Additional Supplementary Files [file 41467_2024_45543_MOESM3_ESM.pdf]

<u>Description of Additional Supplementary Files</u>

Supplementary Movie Legends

Supplementary Movie 1: Simultaneous sensing of eight salvianolic acids. The measurement was carried out as described in Methods. CA, PCA, PA, SAA, RA, LSA, SalA and SalB were simultaneously added to cis with a buffer of 1.5 M KCl, 100 mM MOPS, pH 7.0. The final concentration of CA and SAA was 40 µM, that of PCA was 100 µM, and that of PA, RA, LSA, SalA and SalB wsa 20 µM. During salvianolic acids sensing, a transmembrane potential of +100 mV was continuously applied and highly consistent resistive pulses caused by eight salvianolic acids were clearly observed. All events were identified by machine learning prediction. For demonstration purpose, the movie was played back at a 1.2 x speed of the actual data acquisition.

Supplementary Movie 2: Nanopore sensing of Salvia miltiorrhiza. The measurement was performed using MspA-90PBA in a buffer of 1.5 M KCl, 100 mM MOPS, pH 7.0, as demonstrated in Methods. A transmembrane potential of +100 mV was continually applied and Salvia miltiorrhiza were added to cis with a volume of 20 µL ultrafiltration. Characteristic events of Salvia miltiorrhiza were clearly observed and labeled automatically using machine learning algorithm. The movie was played back at a 1.2 x speed of the actual data acquisition.

Supplementary Movie 3: Nanopore sensing of Rosemary. The measurement was performed using MspA-90PBA in a buffer of 1.5 M KCl, 100 mM MOPS, pH 7.0, as demonstrated in Methods. A volume of 20 µL ultrafiltration of Rosemary were added to cis. A transmembrane potential of +100 mV was continually applied. Characteristic events of Rosemary were observed and identified automatically with machine learning algorithm. The movie was played back at a 1.2 x speed of the actual data acquisition.

Supplementary Movie 4: Nanopore sensing of P. vulgaris. The measurement was performed using MspA-90PBA in a buffer of 1.5 M KCl, 100 mM MOPS, pH 7.0, as demonstrated in Methods. A volume of 20 µL ultrafiltration of P. vulgaris were added to cis. A transmembrane potential of +100 mV was continually applied. Corresponding events were observed and identified using machine learning algorithm. The movie was played back at a 1.2 x speed of the actual data acquisition.
